# Engineering and expression of a human rotavirus candidate vaccine in *Nicotiana benthamiana*

**DOI:** 10.1186/s12985-015-0436-8

**Published:** 2015-12-02

**Authors:** Francisco F. P. G. Pêra, David L. R. Mutepfa, Ayesha M. Khan, Johann H. Els, Sandiswa Mbewana, Alberdina A. A. van Dijk, Edward P. Rybicki, Inga I. Hitzeroth

**Affiliations:** Biopharming Research Unit, Department of Molecular and Cell Biology, University of Cape Town, Rondebosch, Cape Town, South Africa; Biochemistry Division, North-West University, Potchefstroom, South Africa; Institute of Infectious Disease and Molecular Medicine, Faculty of Health Science, University of Cape Town, Cape Town, South Africa

## Abstract

**Background:**

Human rotaviruses are the main cause of severe gastroenteritis in children and are responsible for over 500 000 deaths annually. There are two live rotavirus vaccines currently available, one based on human rotavirus serotype G1P[8], and the other a G1-G4 P[8] pentavalent vaccine. However, the recent emergence of the G9 and other novel rotavirus serotypes in Africa and Asia has prompted fears that current vaccines might not be fully effective against these new varieties.

**Results:**

We report an effort to develop an affordable candidate rotavirus vaccine against the new emerging G9P[6] (RVA/Human-wt/ZAF/GR10924/1999/G9P[6]) strain. The vaccine is based on virus-like particles which are both highly immunogenic and safe. The vaccine candidate was produced in *Nicotiana benthamiana* by transient expression, as plants allow rapid production of antigens at lower costs, without the risk of contamination by animal pathogens. Western blot analysis of plant extracts confirmed the successful expression of two rotavirus capsid proteins, VP2 and VP6. These proteins assembled into VLPs resembling native rotavirus particles when analysed by transmission electron microscopy (TEM). Expression of the rotavirus glycoprotein VP7 and the spike protein VP4 was also tried. However, VP7 expression caused plant wilting during the course of the time trial and expression could never be detected for either protein. We therefore created three fusion proteins adding the antigenic part of VP4 (VP8*) to VP6 in an attempt to produce more appropriately immunogenic particles. Fusion protein expression in tobacco plants was detected by western blot using anti-VP6 and anti-VP4 antibodies, but no regular particles were observed by TEM, even when co-expressed with VP2.

**Conclusion:**

Our results suggest that the rotavirus proteins produced in *N. benthamiana* are candidates for a subunit vaccine specifically for the G9P[6] rotavirus strain. This could be more effective in developing countries, thereby possibly providing a higher overall efficacy for the existing vaccines. The production of rotavirus proteins in plants would probably result in lower manufacturing costs, making it more affordable for developing countries. Further investigation is required to evaluate the immunogenic potential of the VLPs and fusion proteins created in this study.

**Electronic supplementary material:**

The online version of this article (doi:10.1186/s12985-015-0436-8) contains supplementary material, which is available to authorized users.

## Background

Rotavirus (RV) infection has probably been a problem as long as humankind has existed, but the connection between RV as the leading cause of severe diarrhoeal disease and dehydration in children under the age of five worldwide was only made in the 1970s [1]. The disease accounts for one third of hospitalizations for diarrhoea worldwide and results in over 500 000 child deaths per year in under 5-year olds, with mortality greatest in south Asia and sub-Saharan Africa [2–6].

**Fig. 1:**
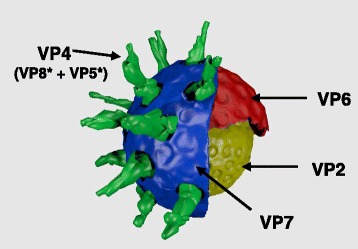
Schematic representation of a rotavirus virion. The virus is composed of an outer capsid (VP7 and VP4), an intermediate capsid (VP6), and an inner capsid (VP2). The outer capsid protein VP4 gets cleaved by proteases located in the host intestinal lumen into VP5* and VP8*. The average size of a rotavirus particle is 70 nm

Rotaviruses are non-enveloped viruses in the family *Reoviridae,* genus *Rotavirus*. Virions are triple-layered and contain a viral genome that consists of 11 segments of double-stranded RNA (dsRNA). These dsRNAs encode six structural proteins (VPs) and six non-structural proteins (NSPs). The capsid is composed of an inner VP2 layer, VP6 forming the intermediate capsid, and an outer layer made of VP7. VP4 forms 60 spikes on the outer surface of the virus (Fig. 1). The VP7 layer is essential for incorporation of VP4 into particles and for the virion to be able to infect a cell. The spike protein VP4 gets cleaved by proteases located in the host intestinal lumen into VP5* and VP8* [7]. VP8* is highly immunogenic and has been proven to elicit high levels of homotypic and heterotypic neutralising antibodies when injected into mice [8]. The neutralizing epitope of VP8* was produced in tobacco plants and has shown to confer protection against bovine rotavirus infection in a mouse model [9].

Rotaviruses are divided into 7 groups (A-G) and four subgroups (I, II, I + II and Non I/II) in group A which are based on the antigenic properties of VP6 [10, 11]. The 2 outer capsid proteins define the dual serotype classification of the viruses, with VP4 (protease-sensitive) defining the P serotype, and VP7 (a glycoprotein) the G serotype [12, 13]. Presently 27 different G-genotypes and 35 P-genotypes have been described, with at least 73 G/P genotype combinations having been found. More recently a classification system that encompasses all 11 RV gene segments has been introduced, and two major genotype (non-G, non-P) constellations termed Wa-like and DS-1-like have been identified [13, 14].

Vaccination is the best control measure to protect against disease, but prior to specific rotavirus vaccine development, the most common serotypes occurring in specific geographic locations need to be determined in order to develop a broadly effective vaccine. There are currently two licensed vaccines available on the market: GlaxoSmithKline’s Rotarix® and Merck’s RotaTeq™. Both of these vaccines are given orally and consist of of live attenuated rotavirus that replicates inside the small intestine. Rotarix® contains the most commonly occurring human rotavirus strain globally, G1P[8]. RotaTeq™ consists of a reassortant collection of 5 viruses comprising 10 bovine rotavirus genes and 5 human rotavirus genes, protecting against the four most prevalent human strains of rotavirus; namely, G1, G2, G3 and G4 and serotype P[4] [15]. A study that reviewed the rotavirus strain types that were found to be circulating in Africa over a 10 year period (1997–2006) revealed that there was an increase in RV strain diversity in Africa, with the recent emergence and spread of the G9 serotype [16–18]. Other studies noted the emergence of G2, G8 and G9 rotaviruses and unusual G and P combinations and a high prevalence of a P[6] VP4 genotype [11, 19]. Emergence of the G9 and novel rotavirus strains in Africa prompted fears that the current vaccines might not protect against these new serotypes, and it is thought that these rotavirus strains need to be taken into account when developing new or better vaccines against rotavirus.

A clinical trial with Rotarix® (G1P[8]) in 2006–2007 showed that this vaccine had a 49.9 % efficacy in Malawi and 76.9 % vaccine efficacy in South Africa [20]. The efficacy of RotaTeq™, the pentavalent rotavirus vaccine, was also only 64.2 % in the first year of life; overall vaccine efficacy against severe rotavirus gastroenteritis was 39 % in developing countries in sub-Saharan Africa, but higher (68–75 %) in developed countries [21, 22]. The efficacy against G9 specifically was 49.7 %. The low efficacy of the two rotavirus vaccines in Africa compared to more developed countries highlights the need to develop a vaccine that will be region-specific and will include prevalent G and P genotypes in the vaccine [23, 24]. Accordingly, in this work we endeavoured to express rotavirus proteins from the new emerging G9P[6] (RVA/Human-wt/ZAF/GR10924/1999/G9P[6]) strain which was recently sequenced and analysed [11, 25].

One disadvantage of live attenuated vaccines remains viral shedding and gene reassortment [26]. An alternative is non-infectious vaccines based on virus-like particles (VLPs): these contain only the viral capsid proteins, with no viral genomic material, and mimic the native rotavirus virion in antigenicity. An important criterion for the use of VLPs as vaccine candidates is their safety. Rotavirus VLPs produced in insect cells and yeast have received attention in recent years as alternative vaccine candidates that are highly immunogenic and safe [27–30].

Plants have recently been utilized as an alternative production system which is suited for the rapid production of antigens at lower costs, and without the risk of contamination by animal pathogens [31]. Various groups have expressed rotavirus capsid proteins in plants. Matsumura (2002) reported bovine rotavirus A VP6 expression in transgenic potato plants. The protein was purified and immunogenicity studies performed: adult mice developed anti-VP6 antibodies in their sera. However, there was no evidence of assembly of the VP6 and immune responses may have been to simple monomers or trimers [32]. Other workers showed assembly of VP6 expressed in *N. benthamiana* via a potato virus X (PVX)-derived vector. The VP6 formed trimers, assembled around VP2 cores, and still assembled when fused to the PVX CP, as protein rods. Once cleaved from PVX CP, the VP6 assembled into icosahedral VLPs [33]. A more recent study showed the successful expression of codon-optimized human rotavirus VP6 in *Chenopodium amaranticolor* using a Beet black scorch virus (BBSV)-mediated expression system with the VP6 gene replacing the CP gene of BBSV. Oral immunization of female BALB/c mice with the plant based VP6 protein induced high titres of anti-VP6 mucosal IgA and serum IgG [34]. The paper did not mention, however, whether or not the VP6 proteins assembled into VLPs. Saldana et al. (2006) successfully expressed VP2 and VP6 in the cytoplasm of fruits from transgenic tomato plants [35]*.* Electron microscopy showed that a small proportion of the particles had assembled into 2/6 VLPs. A protective immune response was detected in mice; however, this may have to some extent been contributed by the non-assembled VPs.

The above studies showed that rotavirus coat proteins can be expressed in relatively high levels in plants; that VP2 and VP6 are capable of forming VLPs in plants, and that these VLPs elicit protective immune responses in animal models. In this work, we report an effort to express several rotavirus proteins in plants via transient agroinfiltration-mediated expression in *N. benthamiana* leaves. These proteins could be considered in the future as candidates for an affordable rotavirus VLP vaccine against the new emerging G9P[6] strain. We investigated the effect of intracellular targeting on expression levels of VP6 by targeting the protein to the ER, apoplastic spaces, chloroplast or cytosol. We also fused the highly immunogenic VP8* or the neutralising epitope of VP8* to VP6 and co-expressed these chimeric proteins together with VP2. We further determined the ability of these proteins to form VLPs by electron microscopy.

## Results and discussion

### Expression of Rotavirus recombinant proteins in tobacco plants

All rotavirus proteins used for this study derive from the G9 P[6] strain which is predominant in South Africa and other sub-Saharan regions [21, 22]. A VLP vaccine targeting this strain would help in alleviating the burden of disease in countries from this region.

*Agrobacterium tumefaciens* cultures containing the recombinant proteins were co-expressed with or without the Tomato spotted wilt virus (TSWV) silencing suppressor protein NSs in *N.benthamiana* to optimise expression in plant cells. NSs is a RNA silencing suppressor protein which inhibits the onset of post-transcriptional gene silencing (PTGS) [36]. Leaf discs were harvested at different time points to determine the optimum day of expression. In an attempt to boost protein yields, we initially tried directing proteins to different intracellular compartments by using several targeting tags. Infiltrating the plants with *Agrobacterium* at different optical densities (ODs) was also done to determine the optimum OD for highest protein expression.

**Fig. 2:**
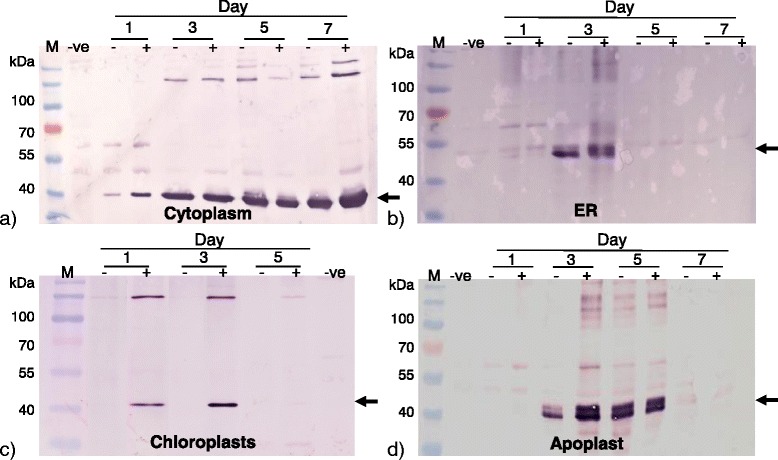
VP6 accumulation in the cytoplasm, ER, chloroplast and apoplast. Different vectors were used to target VP6 to intracellular compartments: **a** pTRAc-VP6 (cytoplasm), **b** pTRAkc-ERH-VP6 (Endoplasmic Reticulum), **c** pTRAkc-rbcs-cTP-VP6 (chloroplast targeting) and **d** pTRAkc-A-VP6 (apoplast). Western blot was performed to access expression levels of rotavirus VP6 protein in plant leaf cell compartments over 7 days. Mouse anti-rotavirus VP6 antibody (1:5000) was used to probe the membranes. (+) and (−) indicates expression with or without silencing suppressor NSs, respectively. Arrows indicate the position of VP6 proteins in the various samples analysed (±42 kDa). M – Pre-stained molecular marker, −ve – plants infiltrated with NSs only

The intermediate capsid protein VP6 was successfully expressed using the plant expression vectors from the pTRA family. The cytoplasm-targeted 40 kDa VP6 was present from day one of the time trial, with increasing protein accumulation during the week-long trial (Fig. 2a). Adding NSs only enhanced cytoplasmic accumulation of VP6 at day 7. Protein accumulation of VP6 in the chloroplasts occurred between days 1 and 3. In contrast to cytoplasmic accumulation, addition of NSs had a considerable effect on accumulation in chloroplasts, as no VP6 was observed in its absence. There was also no detectable protein at days 5 and 7 (Fig. 2c). The apoplast and the ER showed best protein accumulation at day 3 of the time trial and none at all on days 1 and 7. The addition of NSs increased expression levels in the ER and apoplast, especially on day 3 (Fig. 2b,d). The increase in size of the ER-targeted VP6 is probably due to the additional SKDEL and 6xHis sequences present in the pTRAkc-ERH expression vector. VP6 expression was not enhanced by targeting the protein to intracellular compartments, compared to cytoplasmic accumulation (Fig. 2). These results corroborate previous studies where VP6 expression was always higher in the cytoplasm [32, 33].

**Fig. 3:**
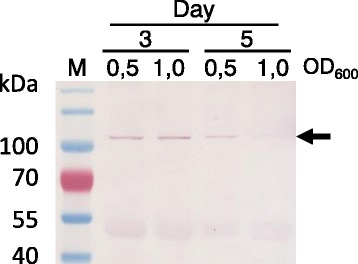
Analysis of pTRAc-HT-VP2 expression with a silencing suppressor (NSs) over time and at different Agrobacterium concentrations. Proteins were detected by western blot using a commercial anti-his antibody (1:2000). M – Pre-stained molecular marker. Arrow indicates expected band size (VP2 ± 100 kDa)

VP2 containing a His tag could only be detected as a ~100 kDa band at low levels in the cytoplasm at 3 and 5 dpi and when using different ODs of *Agrobacterium* for infiltration, and only when co-expressed with the silencing suppressor NSs (Fig. 3). Similar expression levels were seen for VP2 targeted to the ER, chloroplasts and apoplast (data not shown). VP2 expression without a His tag could not be explored due to lack of antibodies for detection. Low expression levels of VP2 in comparison to VP6 have been seen in both plant and insect cell expression studies [26–29].

Expression of the outer capsid proteins VP7 and VP4 was also attempted as they are essential for virion-like immunogenicity and complete particle formation. However, we could never detect expression of either protein on their own in plants, even when different pTRA expression vectors were used (data not shown). Intracellular targeting also did not have an impact on expression. In addition, plants infiltrated with VP7 exhibited yellowing symptoms from day 1 and proceeded to wilt during the time trial. Proceeding with the time trial at a lower temperature of 17 °C in order to reduce the rate of metabolic processes in the plant, and thereby hopefully lower toxicity levels of the protein, was also unsuccessful. Co-infiltrating VP7 with VP2 and VP6 at day 3 was also tried, to see if proteins could be expressed and then immediately assemble with VP2 and VP6 to form VLPs: this also failed.

Difficulties in expressing recombinant VP7 have been previously described for *E. coli* and eukaryote cell expression systems, where VP7 was shown to be toxic for the cells [37, 38]. VP7 contains a natural ER signal sequence that retains it on the ER membrane [39]. It has been suggested that, once in the ER membrane, VP7 causes an increase in the amount of Ca^2+^ inside the ER which leads to a disruption in cell Ca^2+^ homeostasis [40]. These dysregulations in Ca^2+^ concentrations could be responsible for the cytotoxicity and cell death responses observed when expressing VP7 [41]. Despite some studies having reported expression of VP7 in transgenic potatoes and insect cells, it is important to note that these used simian rotavirus VP7 [42] and human group A G1 VP7 [43–45], while our work focused on human rotavirus G9 VP7. Amino acid differences between the proteins in specific domains could be responsible for this effect in *N. benthamiana*. Future studies should focus on identifying these toxic domains and altering them without interfering with protein structure or immunogenicity.

Since VP4 or VP7 could not be expressed in our system, we engineered fusion proteins that would incorporate the highly immunogenic region of VP4 - located in the VP8* segment - into the structural protein VP6: the idea was that this could result in a protein that would assemble into VLPs without the glycoprotein VP7, while still presenting protective VP4 epitopes. In 2008, Istrate et al. showed that VP2 fused to VP8* successfully formed VLPs when expressed in a baculovirus system with VP6 and VP7, and that these particles induced rotavirus-specific serum IgG and IgA in mice [46].

**Fig. 4:**
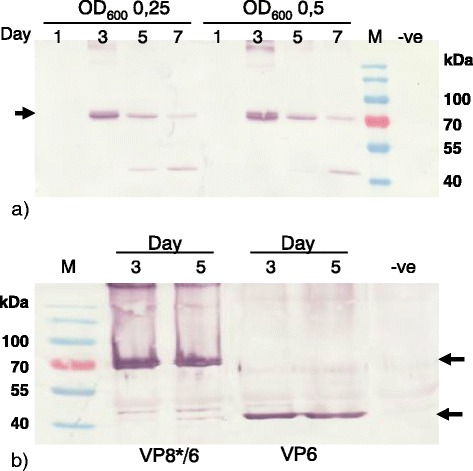
Expression of the chimeric protein VP8*/6. **a** Western blot analysis of pEAQ-VP8*/6 expression in *N. benthamiana* over time at OD_600_.0,25 and 0,5. **b** Western blot of plants infiltrated with either VP8*/6 or VP6 reveals a common band of roughly 45 kDa. Proteins were detected using commercial anti-VP6 antibody (1/5000). M – Pre-stained molecular marker, −ve – plants with infiltration media only. Arrows indicate expected band size (VP8*/6 ± 73 kDa, VP6 ± 42 kDa)

The whole VP8* encoding sequence was inserted upstream of and in-frame with the amino-terminus of the VP6 ORF to create VP8*/6. VP8*/6 expression was confirmed by western blot using a commercial anti-VP6 antibody (1:5000) (Fig. 4a). This resulted in a band at about 75 kDa which corresponds to the theoretical size of the fusion protein VP8*/6 (73 kDa) (Fig. 4a). We were only able to achieve VP8*/6 expression using the binary plant expression vector pEAQ-HT; previous attempts using the plasmids of the pTRA family resulted in low or non-detectable levels of the protein (data not shown). We also noted the appearance of a band of approximately 40 kDa after day 5 (Fig. 4a and b): this corresponds exactly to the size of VP6 and was only detected using anti-VP6 antibodies. We hypothesise that, because VP8*/6 still contains the original VP4 trypsin cleavage site, the 40 kDa band corresponds to VP6 that is produced by cleavage of VP8*/6 by natural trypsin-like proteases present in the leaves of *N.benthamiana*. A study using VP8* fused to VP2 also reported some VP2 degradation of VLPs containing the VP8*-2 fusion protein [47].

**Fig. 5:**
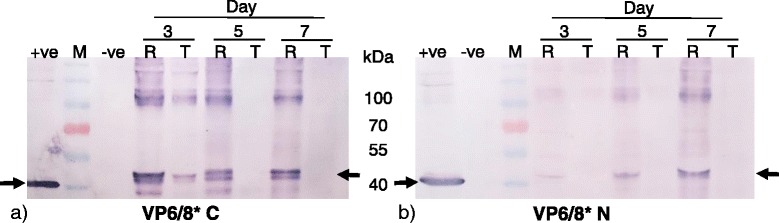
Analysis of VP6/8*(N/C) expression over time using two different expression vectors, pRIC 3.0 and pTRAc-HT. **a** VP6/8*C and **b** VP6/8*N expression analysis. Proteins were detected by immunoblotting using anti-VP6 (1/5000) R – pRIC 3.0 + VP6/8*. T – pTRAc-HT + VP6/8*. M – Pre-stained molecular marker, +ve – pTRAc-VP6. –ve – plants with infiltration media only. Arrows indicate expected band size (VP6/8*(N/C) ± 43 kDa, VP6 ± 42 kDa)

In order to increase chances of particle formation and to prevent protein cleavage, we designed two additional strategies for rotavirus fusion proteins. The 10 amino acid epitope situated at amino acid 1–10 of VP8* has been shown to be highly immunogenic and to elicit specific neutralizing antibodies in mice when expressed in *E.coli* [48]. The VP8* epitope-encoding sequence was fused by means of PCR to either the amino- or carboxyl terminus-encoding sequence of the VP6 ORF to produce VP6/8*N and VP6/8*C respectively. Western blot analysis using anti-VP6 antibodies confirmed the presence of both VP6/8*N and -C versions in the cytoplasm of plant cells (Fig. 5a and b). Curiously, VP6/8*C appeared to be expressed more rapidly than VP6/8*N, with highest expression levels at 3 d.p.i and 7 d.p.i., respectively. This shows that the positioning of the epitope may have an impact on protein accumulation.

**Fig. 6:**
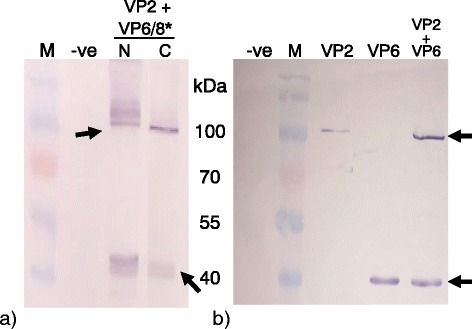
Co-expression of VP2 with VP6 and VP6/8*(N/C). **a** Western blot analysis of crude extract of plants co-expressed with VP2 + VP6/8*. **b** Western blot of crude extract of plants co-expressed with VP2 + VP6. VP2 appears to be in higher amounts when co-expressed with VP6. Proteins were detected using anti-VP6 antibody (1/5000) and anti-histidine (1/2000). M – Pre-stained molecular marker, −ve – plants with infiltration media only. N – VP8* epitope fused to the amino terminus of VP6. C – VP8* epitope fused to the carboxy terminus of VP6. Arrows indicate expected band size (VP6*/8(N/C) ± 43 kDa, VP2 ± 100 kDa, VP6 ± 42 kDa)

Since cytoplasmic accumulation was best for both rotavirus protein expression and extraction efficiency, all co-expression work was done exclusively with proteins accumulating in the cytoplasm. VP2 is a scaffolding protein that is essential in particle formation; therefore, co-expression of VP2 with VP6, VP8*/6, VP6/8*N and VP6/8*C was investigated. Co-expressed proteins were detected by western blot with anti-His and anti-VP6 antibodies. All proteins were detected from 3 days post infiltration (Fig. 6a and b). VP2 expression levels appeared to be higher when co-expressed with VP6 then when expressed alone: our hypothesis is that VP6 must be providing protection by assembling around the VP2 core as in natural virion morphogenesis, and preventing degradation.

To confirm VLP formation, crude extracts of infiltrated plants were fractionated by sucrose gradient centrifugation. Crude extracts were centrifuged on 10 to 60 % sucrose gradients and aliquots from each fraction analysed by immunoblots probed with mouse anti-VP6 and/or mouse anti-His antibodies (Additional file 1: Figure S1). All VPs and derivatives were found in the 20–30 % sucrose zone (data not shown).

### Electron microscopy

**Fig. 7:**
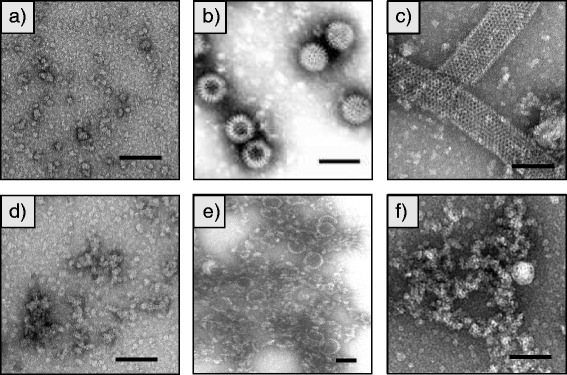
Detection of rotavirus VLPs by transmission electron microscopy. **a** Negative control, plants infiltrated with infiltration media only. **b** Live rotavirus particles (Photo Credit: F.P. Williams, U.S.) (Environmental Protection Agency (EPA), 2013). **c** VP6. **d** VP8*/6. **e** HT-VP2 + VP6. **f** HT-VP2 + VP8*/6. Grids were coated with anti-VP6 (1/500). All plants were harvested at 3 dpi. Bars represent 100 nm

Transmission electron microscopy was used to analyse particles formed by single and co-expressed recombinant proteins. VP6 alone assembled into helical and tubular structures as previously described [49, 50] (Fig. 7c). However, when co-expressed with VP2, spherical particles resembling the natural virions were seen, in both crude (Fig. 7e) and sucrose gradient-purified preparations (Additional file 2: Figure S2). The assembly of VP2/6 particles has been well documented in the insect-cell expression system [33, 51]. VP2/6 VLPs have been shown to elicit immune responses that are protective against rotavirus infections in mice [52, 53], and gnobiotic piglets [54]. Particles comprised of VP2/6/7 and 2/4/6/7 have also been expressed in insect cells; these were generally more immunogenic and prevented rotavirus infections in different animal models [55, 56]. However, difficulty in obtaining large quantities of RV-VLPs containing the outer surface proteins VP4 and VP7 was experienced in nearly every study. Accordingly, we experimented with creating fusion proteins based on the structural properties of VP6 and the immunogenicity of VP4.

Unfortunately, despite numerous attempts, no VLPs were seen by EM for crude or purified extracts of plants expressing VP8*/6 (Fig. 7d and f), VP6/8*N or VP6/8*C (data not shown) single and co-expressed with VP2. Single expression of VP8*/6, VP6/8*N or VP6/8*C only resulted in the appearance of amorphous protein aggregates when compared with plants infiltrated with infiltration media only (negative control). We hypothesise that both the full VP8*sequence and the smaller VP8* epitope must be impeding VP6 trimerisation and particle formation. Even though few studies have been made on the structural impact of fusions with structural proteins, there have been reports of conformational changes caused by the simple addition of polyhistidine tags [57, 58]. Moreover, a recent study on multi-epitope fusions showed that polypeptides with small differences in sequence still maintained similar levels of antigenicity, but protein folding and assembly were drastically affected [59]. Thus, the fusion proteins created in this study could still be highly immunogenic even if they do not form VLPs. Animal experiments still have to be performed to analyse the ability of these chimeric proteins as single recombinant subunit vaccines that are cheaper, safer and adapted to the South African strain G9[P6].

## Conclusion

Efforts to help alleviate the burden of rotavirus disease in sub-Saharan Africa and other developing countries have increased significantly in recent years. In this study, we evaluated the possibility of producing rotavirus VLPs using a plant expression system to produce a vaccine specifically adapted to the sub-Saharan African regions. We had partial success in demonstrating the capacity of the transient plant expression system to express specific rotavirus proteins. Despite the fact that no VLPs were observed for our fusion proteins, expression was detected for all chimeric proteins engineered, illustrating the versatility of plant-based systems. While this work is preliminary, we believe that it will serve as a solid basis for future studies on plant-made rotavirus vaccines for Africa.

## Methods

### Plasmid construction

VP2, VP4, VP6 and VP7 sequences (GenBank accession No: FJ183354, FJ183356, FJ183358 and FJ183360) from human rotavirus G9P[6] were plant codon optimised by Geneart, Germany (GenBank accession No: KT931666, KT931667, KT931668 and KT931669 respectively) [25]. The DNA was transformed in DH5-α chemically competent E. coli cells (E. cloni™, Lucigen) as per manufacturer’s instructions. DNA was extracted and genes were transformed into plant expression vectors, pTRAc (cytoplasm), pTRAkc-rbcs-cTP (chloroplast targeting), and pTRAkc-ERH (endoplasmic reticulum targeting) (Kindly supplied by R. Fischer, Fraunhofer Institute for Molecular Biology and Applied Ecology, IME, Germany) [60]. VP2, VP4 and VP6 cDNA was digested with NcoI/XhoI while VP7 was cut with AflIII/XhoI to clone into pTRAc and pTRAkc-ERH. pTRAc-HT was used for VP2, VP4 and VP7 because no specific antibodies were available; pTRAc-HT contains a 6Xhis-tag sequence upstream of the multiple cloning site (MCS), which allows for a 6Xhis-tag attached at the N-terminus of the protein of interest. For cloning in pTRAkc-ERH, a NotI restriction enzyme site was added to replace the stop codon of each the four rotavirus cDNA by PCR amplification utilising the primers as detailed in Additional file 3: Table S1. An additional vector, pTRAkc-A (apoplast), was derived from pTRAkc-ERH by cutting the vector with NcoI and XhoI thereby removing the histidine tag and the ER retention signal (SEKDEL) sequence and resulting in proteins that are targeted to the apoplast. For cloning into pTRAkc-rbcs-cTP, cDNA and vector were digested with MluI/XhoI. DNAs were sequenced to verify fidelity of the PCR reaction.

### Fusion protein design

To create VP8*/6, primers were used to introduce by PCR, a NcoI site at the 3′ end of the VP8* sequence located in the VP4 gene. VP6 was then cut with NcoI and ligated to VP8*; recombinant constructs were further modified by PCR to include an AgeI site on the 5′end thus allowing for cloning into the pEAQ-HT vector (Additional file 3: Table S1) [61]. For VP6/8*N and VP6/8*C, specially designed primers were used to create the VP8*epitope on the desired terminus of the VP6 gene (Additional file 3: Table S1); each primer overlapped with 18–21 nucleotides of VP6. Forward primers added a NcoI site upstream of the VP6 whereas a XhoI site was supplemented on the 3′ end of the gene by the reverse primers. These sites allowed for cloning into the plant expression vectors pTRAc and pRIC 3.0. pRIC 3.0 is a self-replicating vector previously engineered and shown to increase recombinant protein expression in *N. benthamiana* [62].

*Agrobacterium tumefaciens* GV3101::pMP90RK was used with rotavirus pTRA-VP and pRIC-VP constructs whereas *A. tumefaciens* LBA4404 was used with the pEAQ-VP constructs. Electrocompetent Agrobacterium was mixed with 300 ng of recombinant DNA in a 0.1 cm electrogap cuvette (BioRadTM) then electroporated in a GenePulser (BioRad) under the following settings: 1.8 kV, 25 μF and 200 Ώ [63]. Incubation was allowed for 1 h at 27 °C in 900 μl of LB before plating on LA plates containing 30 μg/ml kanamycin (kan), 50 μg/ml rifampicin (rif), 50 μg/ml carbenicillin (carb) was added to cultures electroporated with pTRA-VP constructs and rif and kan was added to pEAQ-VP constructs.

### Recombinant *Agrobacterium* infiltration

Agroinfiltrations were carried out as described in Maclean et al. 2007 [60]. Briefly *A. tumefeciens* LBA 4404 (pEAQ-VPs) and *A. tumefeciens* GV3101(pMP90RK) were grown in LB with respective antibiotics and then harvested and re-suspended in induction media with antibiotics (LB, 10 mM 2-(N-morpholino) ethanesulphonic acid MES, 2 mM MgSO4, 20 μM acetosyringone pH 5.6), and grown overnight at 27 °C. Overnight cultures were then spun down and resuspended in infiltration medium (10 mM MES, 10 mM MgCl2, 3 % sucrose, pH 5.6, 200 μM acetosyringone and sterile water) to reach the desired OD_600_ at 22 °C for 2–3 h [63]. Three week old wild type *N. benthamiana* plants were used for agroinfiltration. The VP proteins were each infiltrated in separate plants either by vacuum infiltration of whole plants or injection of recombinant *Agrobacterium* into the abaxial air spaces on the ventral side of plant leaves. Recombinant *Agrobacterium* was infiltrated either with or without the silencing suppressor LBA 4404 (pBIN-NSs). One plant was used per construct over a seven day time trial and each experiment was done in triplicate.

### Protein extraction and detection

Three leaf discs or whole leaves were harvested for each construct and ground in liquid nitrogen. Ground leaf matter was re-suspended in 1x sterile PBS containing Complete Protease Inhibitor (EDTA-free; Roche) at a ratio of 3:1 (buffer volume : plant weight). This was then centrifuged for 5 min at 25,861 × g and the pellets (plant leaf matter) discarded. An additional extraction procedure was carried out on the proteins that were targeted to the apoplast utilising the pTRAkc-A vector. Whole leaves from each extraction day were vacuum infiltrated with sterile PBS containing Complete Protease Inhibitor. Individual plant leaves were suspended in PBS and put under a vacuum at 100 mbar for 10 min in a vacuum tank. The leaves were then rolled and placed in spin columns (similar to Qiagen spin columns) which were placed in 2 ml Eppendorf tubes and centrifuged at 2448 × g for 15 min and the filtrate was collected.

For western blot analysis, extracts were mixed with 5X SDS-PAGE loading buffer (2 % SDS, 0.1 M Tris–HCl pH 7.5, 20 mM EDTA, 50 % Glycerol, 4 % β-mercaptoethanol, 0.02 % bromophenol blue) and boiled for 2 min at 95 °C, 30uL of samples were loaded and separated on 12 % SDS-PAGE gels and transferred onto nitrocellulose membrane by semi-dry electroblotting. VP proteins were detected using mouse anti-rotavirus VP6 antibody (US Biologicals) (1:5000), mouse anti-histidine tag antibody (anti-His) (Sigma®) (1:2000), followed by goat anti-mouse alkaline phosphate conjugated antibodies (1:10 000 Sigma).

### Sucrose gradient purification of rotavirus VLPs

Plant protein extracts were initially filtered through miracloth to remove solid plant matter. Sucrose gradients from 10 to 60 % sucrose were set up in 40 ml tubes each by creating six layers of 5 ml of sucrose dissolved in sterile PBS (pH 7.4). Clarified protein samples in 5 to 10 ml volumes were then loaded on top of each gradient column. Ultracentrifugation at 150 000 × g (SWTi28 swinging bucket rotor, Beckman Coulter) was carried out at 4 °C for 1 h 30 min. At the end of the centrifugation, 2 ml fractions were collected from the bottom of each column by tube puncture. Dot blots were then performed to determine fractions with proteins of interest. For each fraction, 1 μl of sample was loaded in a grid on a nitrocellulose membrane, which was then blocked with BSA blocking buffer. Western blot analysis was then performed as usual. Proteins were probed with mouse anti-VP6 antibody (1:5000) for VP6 or anti-His for VP2 (1:2000).

### Electron microscopy

To determine whether expressed proteins assembled into VLPs, transmission electron microscopy (TEM) of immuno-trapped particles was performed. Glow discharged carbon/copper grids were placed on 20 μl of mouse anti-rotavirus VP6 antibody (1:500) for 5 min and then washed 3 times with sterile distilled water. Grids were then placed on 10 μl of the protein extracts and left for 2 min before being washed 3 times again with sterile distilled water. Finally, grids were floated on 20 μl of 2 % uranyl acetate for 1 min before viewing under a TEM (Zeiss 912 OMEGA Energy Filter Transmission Electron Microscope, University of Cape Town).

For samples isolated from sucrose gradients, the sucrose first had to be removed by dialysis before immune-trapping on the copper grids. The sucrose fractions were placed in 10 000 MW dialysis cassettes and dialysed in sterile PBS containing 0.4 M NaCl for 4 h before exchanging the buffer and leaving it overnight at 4 °C with stirring.

## Additional files

Additional file 1: Figure S1. Analysis of sucrose density gradient purified VP2/6. A) Western blot analysis of VP2/6 fractions using mouse anti-VP6 (1:5000) and anti-His (1:2000). B) SDS-PAGE coomassie stained gel of fractions. -ve – Fraction 16 of plants infiltrated with silencing suppressor only and sucrose gradient purified. +ve – VP6 expressed in insect cells. Arrows indicate protein band size. (PPTX 1090 kb) Additional file 2: Figure S2. Transmission electron micrograph of sucrose density gradient purified VP2/6 particles. Purified VP2/6 fractions were pooled together and dialysed in high salt PBS to remove sucrose before viewing on a transmission electron microscope. Most of the VLPs remained intact but some appeared to have lost shape probably as a result of deformation due to the conditions on the EM grid. Samples were captured with mouse-anti VP6 antibody (1/500). Bar represents 100 nm. (PPTX 1531 kb) Additional file 3: Table S1. List of primer sequences used for rotavirus chimeric protein formation. (PPTX 48 kb)
