# Supplementary material for: Engineering and expression of a human rotavirus candidate vaccine in Nicotiana benthamiana
Source: Virol J. 2015 Dec 2;12:205. doi: 10.1186/s12985-015-0436-8 (PMC4667453; doi:10.1186/s12985-015-0436-8)
Supplement: Additional file 1: Figure S1. — Analysis of sucrose density gradient purified VP2/6. A) Western blot analysis of VP2/6 fractions using mouse anti-VP6 (1:5000) and anti-His (1:2000). B) SDS-PAGE coomassie stained gel of fractions. -ve – Fraction 16 of plants infiltrated with silencing suppressor only and sucrose gradient purified. +ve – VP6 expressed in insect cells. Arrows indicate protein band size. (PPTX 1090 kb) [file 12985_2015_436_MOESM1_ESM.pptx]

## Slide 1
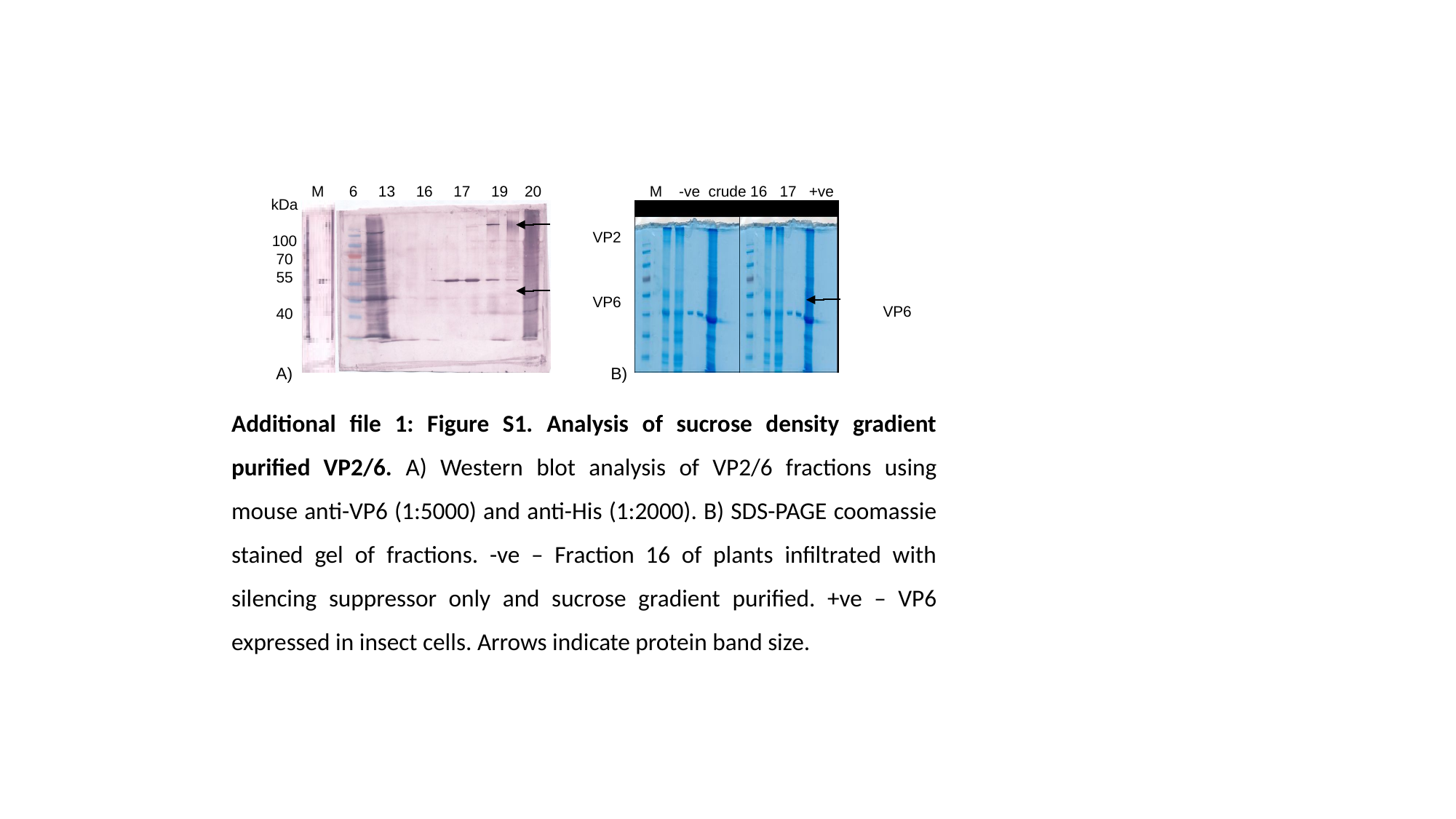

M 6 13 16 17 19 20
kDa
10070
55
40
VP2
VP6
 M -ve crude 16 17 +ve
VP6
A)
B)
Additional file 1: Figure S1. Analysis of sucrose density gradient purified VP2/6. A) Western blot analysis of VP2/6 fractions using mouse anti-VP6 (1:5000) and anti-His (1:2000). B) SDS-PAGE coomassie stained gel of fractions. -ve – Fraction 16 of plants infiltrated with silencing suppressor only and sucrose gradient purified. +ve – VP6 expressed in insect cells. Arrows indicate protein band size.
