# Supplementary material for: Engineering and expression of a human rotavirus candidate vaccine in Nicotiana benthamiana
Source: Virol J. 2015 Dec 2;12:205. doi: 10.1186/s12985-015-0436-8 (PMC4667453; doi:10.1186/s12985-015-0436-8)
Supplement: Additional file 2: Figure S2. — Transmission electron micrograph of sucrose density gradient purified VP2/6 particles. Purified VP2/6 fractions were pooled together and dialysed in high salt PBS to remove sucrose before viewing on a transmission electron microscope. Most of the VLPs remained intact but some appeared to have lost shape probably as a result of deformation due to the conditions on the EM grid. Samples were captured with mouse-anti VP6 antibody (1/500). Bar represents 100 nm. (PPTX 1531 kb) [file 12985_2015_436_MOESM2_ESM.pptx]

## Slide 1
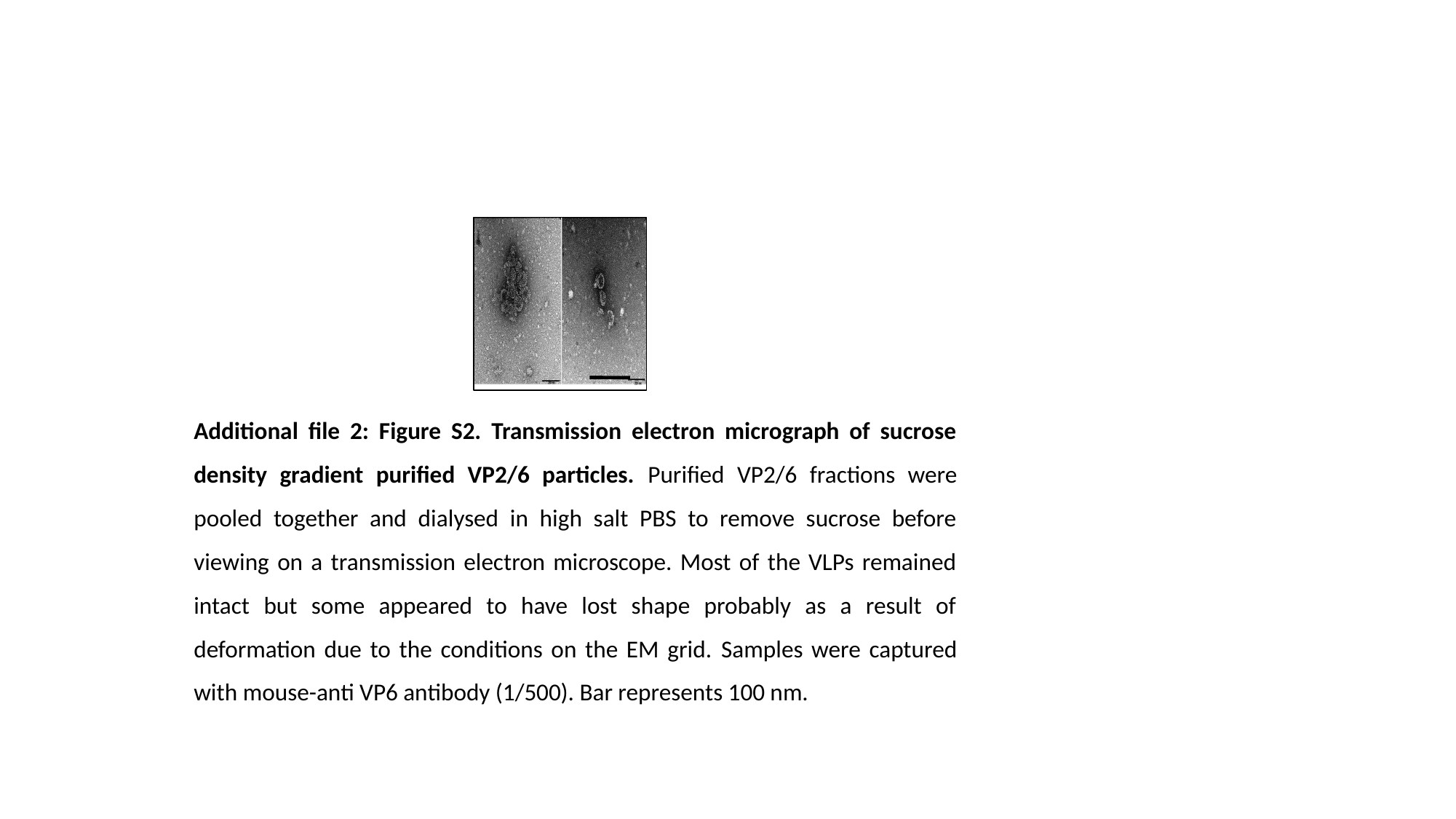

Additional file 2: Figure S2. Transmission electron micrograph of sucrose density gradient purified VP2/6 particles. Purified VP2/6 fractions were pooled together and dialysed in high salt PBS to remove sucrose before viewing on a transmission electron microscope. Most of the VLPs remained intact but some appeared to have lost shape probably as a result of deformation due to the conditions on the EM grid. Samples were captured with mouse-anti VP6 antibody (1/500). Bar represents 100 nm.
