# Supplementary material for: Engineering and expression of a human rotavirus candidate vaccine in Nicotiana benthamiana
Source: Virol J. 2015 Dec 2;12:205. doi: 10.1186/s12985-015-0436-8 (PMC4667453; doi:10.1186/s12985-015-0436-8)
Supplement: Additional file 3: Table S1. — List of primer sequences used for rotavirus chimeric protein formation. (PPTX 48 kb) [file 12985_2015_436_MOESM3_ESM.pptx]

## Slide 1
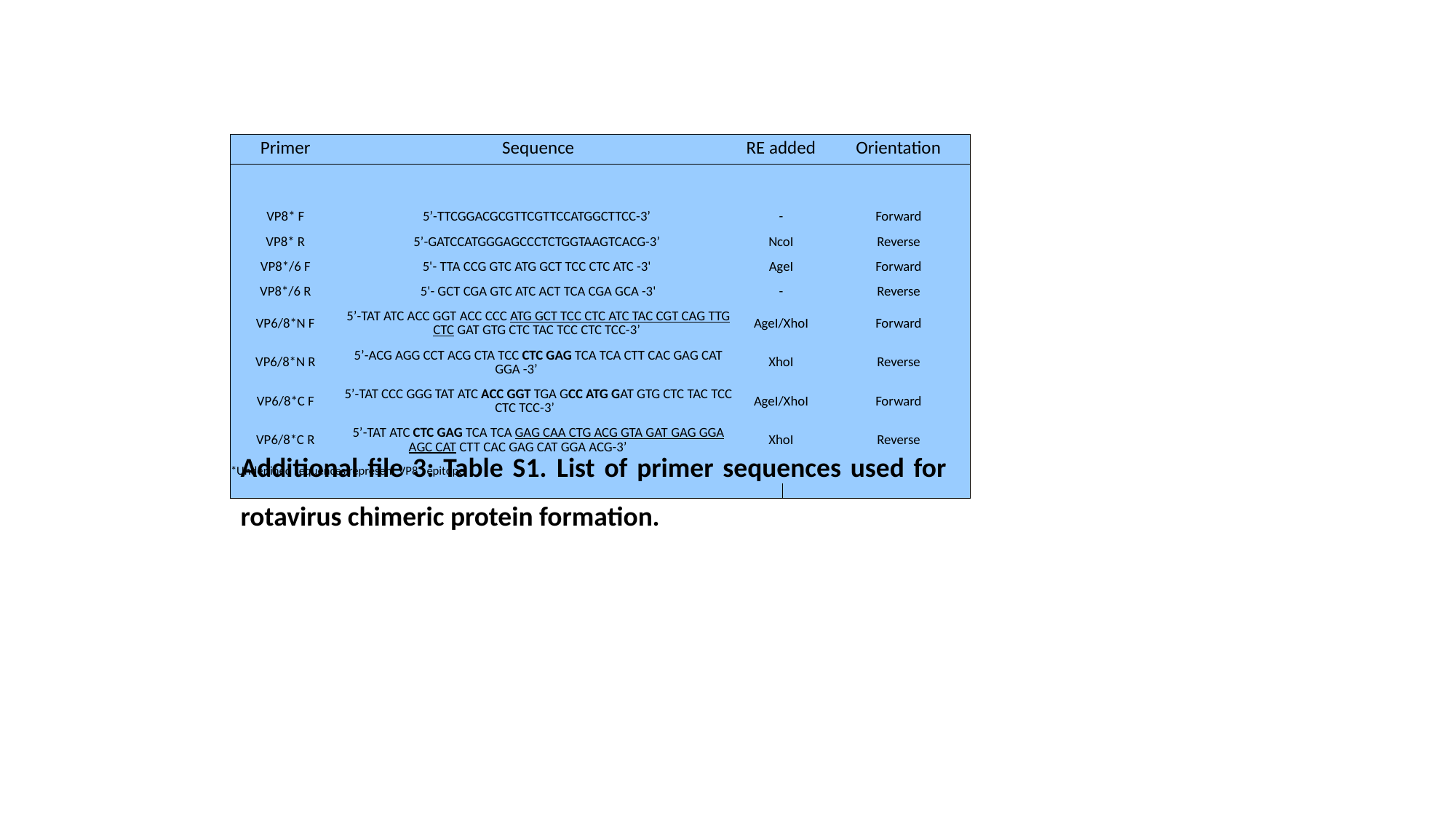

| Primer | Sequence | RE added | | | Orientation |
| --- | --- | --- | --- | --- | --- |
| | | | | | |
| VP8\* F | 5’-TTCGGACGCGTTCGTTCCATGGCTTCC-3’ | - | | | Forward |
| VP8\* R | 5’-GATCCATGGGAGCCCTCTGGTAAGTCACG-3’ | NcoI | | | Reverse |
| VP8\*/6 F | 5'- TTA CCG GTC ATG GCT TCC CTC ATC -3' | AgeI | | | Forward |
| VP8\*/6 R | 5'- GCT CGA GTC ATC ACT TCA CGA GCA -3' | - | | | Reverse |
| VP6/8\*N F | 5’-TAT ATC ACC GGT ACC CCC ATG GCT TCC CTC ATC TAC CGT CAG TTG CTC GAT GTG CTC TAC TCC CTC TCC-3’ | AgeI/XhoI | | | Forward |
| VP6/8\*N R | 5’-ACG AGG CCT ACG CTA TCC CTC GAG TCA TCA CTT CAC GAG CAT GGA -3’ | XhoI | | | Reverse |
| VP6/8\*C F | 5’-TAT CCC GGG TAT ATC ACC GGT TGA GCC ATG GAT GTG CTC TAC TCC CTC TCC-3’ | AgeI/XhoI | | | Forward |
| VP6/8\*C R | 5’-TAT ATC CTC GAG TCA TCA GAG CAA CTG ACG GTA GAT GAG GGA AGC CAT CTT CAC GAG CAT GGA ACG-3’ | XhoI | | | Reverse |
| \*Underlined sequences represent VP8\* epitope | | | | | |
| | | | | | |
Additional file 3: Table S1. List of primer sequences used for rotavirus chimeric protein formation.
